# Mechanical Motion and Color Change of Humidity-Responsive Cellulose Nanocrystal Films from Sunflower Pith

**DOI:** 10.3390/polym16223199

**Published:** 2024-11-18

**Authors:** Shujie Wang, Yanan Liu, Zhengkun Tao, Yang Li, Jie Jiang, Ke Zheng

**Affiliations:** 1 Biomass Molecular Engineering Center and Department of Materials Science and Engineering, School of Forestry and Landscape Architecture, Anhui Agricultural University, 130 West Changjiang Road, Hefei 230036, China; jiangdong412@stu.ahau.edu.cn (S.W.); liuyanan@stu.ahau.edu.cn (Y.L.); 19856265392@163.com (Z.T.); 23720191@stu.ahau.edu.cn (Y.L.); 2 School of Resources and Environmental Engineering, Jiangsu University of Technology, Changzhou 213001, China

**Keywords:** sunflower pith, nanocellulose, humidity-responsive, shape deformation, structural color

## Abstract

Nanocellulose has prompted extensive exploration of its applications in advanced functional materials, especially humidity-responsive materials. However, the sunflower pith (SP), a unique agricultural by-product with high cellulose and pectin content, is always ignored and wasted. This work applied sulfuric acid hydrolysis and sonication to sunflower pith to obtain nanocellulose and construct film materials with humidity-responsive properties. The SP nanoparticle (SP-NP) suspension could form a transparent film with stacked layers of laminated structure. Due to the tightly layered structure and expansion confinement effect, when humidity increases, the SP-NP film responds rapidly in just 0.5 s and completes a full flipping cycle in 4 s, demonstrating its excellent humidity-responsive capability. After removing hemicellulose and lignin, the SP cellulose nanocrystals (SPC-NC) could self-assemble into a chiral nematic structure in the film, displaying various structural colors based on different sonication times. The color of the SPC-NC film dynamically adjusted with changes in ambient humidity, exhibiting both functionality and aesthetics. This research provides a new perspective on the high-value utilization of sunflower pith while establishing a practical foundation for developing novel responsive cellulose-based materials.

## 1. Introduction

Cellulose, the primary component of natural lignocellulosic biomass [1], offers advantages such as abundance, renewability, and environmental friendliness [2]. Notably, agricultural by-products rich in cellulose, such as straw, rice husk, and corn cobs, are often subjected to incineration, resulting in inefficient utilization [3,4]. A feasible strategy for functionalizing cellulose materials is the hierarchical delamination of cellulose feedstocks to produce nanocellulose. Currently, methods such as pure mechanical treatment, acid hydrolysis [5,6], refined mechanical processing [7], and TEMPO oxidation [8,9] have been demonstrated to efficiently produce nanocellulose with high specific surface area, low thermal expansion coefficient, and excellent mechanical strength. Furthermore, these nanocellulose materials can be used to construct cellulose-based functional materials with outstanding optical and mechanical properties [10,11].

Sunflower (*Helianthus annuus*) [12], a member of the Asteraceae family, has a pith rich in cellulose [13], comprising approximately three-quarters of the outer stem volume. Sunflower pith exhibits a higher cellulose content than the pith composition of sorghum and corn stems [14]. A comprehensive assessment reveals that sunflower pith contains relatively high proportions of cellulose, pectin, and ash, while hemicellulose and lignin contents are comparatively lower. This unique composition endows sunflower pith with significant potential for applications in functional materials, particularly in the field of sustainable material development [15,16]. Sunflower pith has already demonstrated promise in various applications, including solar evaporation systems [13,17], adsorptive materials [18], and the extraction of pectin and glucose [19]. However, despite these research efforts providing valuable insights into the comprehensive development of sunflower pith, its exploration for broader applications remains relatively limited compared to other lignocellulosic feedstocks, such as food packaging [20,21] and electrode materials [22]. This indicates that there is still much untapped potential in the application development of sunflower pith.

Nanocellulose exhibits exceptional water absorption capacity and swelling ability owing to its large specific surface area and abundance of hydroxyl groups, significantly enhancing its responsiveness to changes in humidity [23,24,25]. Researchers have combined the humidity responsiveness of cellulose with the shape, structure, optical properties, and electrical conductivity of composite materials, leading to the development of nanocellulose-based humidity-responsive devices. These advancements have driven the application of cellulose-based materials in actuators, sensors, soft robotics, and wearable devices [26,27]. Most humidity-responsive cellulose films are composed of multilayer structures, where asymmetry in water absorption and swelling on different surfaces causes the film to bend when exposed to humidity fluctuations [28,29,30]. Current research efforts primarily focus on constructing humidity actuators or high-humidity-sensitive papers and films using nanocellulose in conjunction with other hydrophilic polymers [26]. In contrast, studies on single-layer humidity-responsive cellulose actuators remain scarce.

Meanwhile, cellulose nanocrystals (CNCs), which possess a short rod-like morphology, can self-assemble into chiral nematic structures in aqueous phases [31,32,33]. This unique chiral structure can be retained in cellulose films after water evaporation. When the pitch of the chiral structure falls within a specific range, it selectively reflects visible light at certain wavelengths, exhibiting vibrant structural colors [31,34]. The structural color of the films can be modulated through various methods, including controlling evaporation time [35], applying magnetic fields [36], adjusting ultrasound time [37], or introducing electrolyte salts [38,39]. At the same time, researchers are exploring methods such as chemical modification [40,41], cross-linking [42], and compounding [43,44,45] to enhance the environmental adaptability and stability of CNC-based films to meet a broader range of application needs. Due to the high hydrophilicity of nanocellulose, these films also undergo reversible swelling or shrinkage in response to varying humidity levels. As the chiral pitch changes, the structural color of the film shifts accordingly, either blue-shifting or red-shifting. Research on humidity-responsive structural color cellulose films derived from agricultural waste, particularly sunflower pith, remains notably underexplored.

In this study, SP with high cellulose content was utilized as the raw material, and sulfuric acid hydrolysis was applied separately to both SP and the purified SP cellulose (SPC) to prepare nanocellulose-based film materials through hydrolysis and subsequent drying. The effects of the purification process on the compositional content of the pith were investigated. Subsequently, we examined the influence of ultrasonic treatment time on the morphology and size of the CNCs. The study revealed that cellulose-based films, prepared by direct sulfuric acid hydrolysis of SP, exhibited reversible deformation under humidity-driven conditions. The CNC exfoliated from the hydrolyzed SPC could self-assemble to a chiral nematic structure, and the resulting films displayed humidity-responsive structural coloration. Furthermore, we explored the molecular-level interaction mechanisms between water molecules and CNC in the two kinds of films. This research provides a new perspective on the valorization of agricultural waste and lays the theoretical foundation for developing and implementing responsive cellulose-based materials.

## 2. Materials and Methods

### 2.1. Materials

The sunflower stalks used in this study were purchased from agricultural product company (Chifeng, Inner Mongolia). Sulfuric acid solution (AR, 98%), sodium hydroxide (AR, 96%), sodium hypochlorite (AR, 98%), and regenerated cellulose dialysis membranes were procured from Sinopharm Chemical Reagent (Shanghai, China). All chemicals were used as received without further purification. Deionized water was prepared using a Milli-Q water purification system.

### 2.2. Purification of SPC from SP

In this study, cellulose was systematically extracted from SP using the sodium hydroxide-sodium chlorite (NaOH-NaClO_2_) bleaching method, following a well-established protocol from the literature [46,47]. Initially, SP of 20 g was subjected to a pretreatment process using a mixture of benzene and ethanol in a 2:1 ratio. After treatment, the mixture was vacuum-filtered, thoroughly washed with water, and air-dried. The powdered material was then treated with a 7% sodium hydroxide solution in a water bath at 90 °C for 2 h. The residue was neutralized and dried upon completion, and the entire process was repeated twice to ensure thoroughness.

For the bleaching step, the powder was mixed with a 1.5% (*w*/*v*) sodium chlorite solution in a 1:25 ratio, and the pH of the mixture was adjusted to 3–4. The mixture was stirred in a water bath at 80 °C for 2 h. After bleaching, the mixture was repeatedly washed until neutral, followed by air drying. Finally, the dried SPC was pulverized using a grinder and sieved for further applications.

### 2.3. Preparation of SP Nanoparticle Composites (SP-NP) and SPC Nanocellulose (SPC-NC)

The SP or SPC samples were cut into small segments (a few centimeters in length) and then pulverized using a disperser (Model FZ102, Keheng Industrial Development, Shanghai, China) operating at a speed of 1400 rpm to generate fine microfibers. The extraction of nanocellulose was carried out through sulfuric acid hydrolysis [48,49]. Specifically, 10 g of the pulverized SP or SPC was reacted with 160 mL of 64% (*w*/*w*) sulfuric acid, preheated to 50 °C, for 2 h. The reaction was terminated by adding 800 mL of deionized water. The resulting suspension was centrifuged at 10,000 rpm for 10 min and dialyzed in deionized water to remove residual chemicals. The pH of the solution was adjusted to 5.5, and the concentration of modified SP or SPC suspension was determined by gravimetric analysis. The obtained product suspensions underwent sonication at different times. The SP-NP and SPC-NC were separated from the mixtures by centrifugation.

### 2.4. Preparation of SP-NP Films and SPC-NC Films

To prepare SP-NP film, 8 mL of a 0.2% SP-NP suspension was evenly dispersed in a plastic petri dish with a diameter of 3.5 cm and allowed to air-dry at room temperature for seven days, forming a nanocellulose film. Five portions of 10 mL SPC-NC suspensions sonicated at different times were placed in polystyrene petri dishes and air-dried under ambient conditions for seven days.

### 2.5. Characterization Methods

The component contents of SP and SPC were measured according to the standard methods set by the National Renewable Energy Laboratory (NREL). Specific contents of glucose, xylose, arabinose, and galactose were accurately determined using a Dionex ICS-5000 ion chromatograph (Thermo Fisher Scientific, Waltham, MA, USA). The Fourier Transform Infrared (FT-IR) spectra of SP and SPC were recorded using a Nicolet 6670 FT-IR spectrometer (Thermo Fisher Scientific, Waltham, MA, USA) to elucidate their molecular structure and functional group information. The X-ray diffraction (XRD) patterns of SP and SPC were obtained using an XD-6 X-ray diffractometer (Bruker, Beijing, China). The transmittance and ultraviolet shielding efficiency of the SP-NP films were quantitatively measured in the wavelength range of 300–800 nm using a TU-1950 UV-Vis spectrophotometer (PERSEE, Beijing, China). A JEOL JSM-7800 scanning electron microscope (JEOL Ltd., Tokyo, Japan) was employed to observe the surface and cross-sectional morphology of the films, operating at an accelerating voltage of 5 kV. All samples were pre-coated with a 5-nanometer thick gold layer to prevent charge accumulation that could affect image quality. A Bruker Dimension Icon4-Sys atomic force microscope (Bruker, Beijing, China) was used to investigate the fine morphology and size distribution of the nanocellulose. An Olympus BX 51-P (Olympus, Tokyo, Japan) polarized light microscope, equipped with phase contrast and polarized imaging capabilities, was used to confirm the formation and characteristics of the liquid crystal phase of SPC-NC. A USB 4000 spectrofluorometer (Ocean Optics, Orlando, FL, USA) was applied to analyze the optical properties of SPC-NC films in the 200–1000 nm wavelength range. Dynamic light scattering (DLS) measurements were performed using a DelsaMax PRO system (Beckman Coulter, Shanghai, China) to determine the zeta potential values and particle diameter distributions of SP-NP and SPC-NC in aqueous solution. To extract nanoparticles and nanocrystals, a 2 wt% nanosolution was treated with a Qsonica Q500 ultrasonic probe (Qsonica, Newtown, CT, USA) in 100 mL of distilled water at an amplitude of 36 µm (30% of the maximum amplitude output of the ultrasonic probe, which is 120 µm) and a frequency of 20 kHz for a desired duration. Under ambient temperature conditions, the sample underwent three measurements with a high-precision contact angle goniometer OCA-15EC (Dataphysics, Stuttgart, Germany), and the average value was recorded as the final contact angle (CA).

### 2.6. Data Analysis

The AFM images of SP-NP and SPC-NC were processed using Nanoscope Analysis 1.8 software (Brucker, Beijing, China). All experiments were conducted in triplicate at a minimum, and the aspect ratio of the data was quantified using ImageJ2 software. Finally, the graphs were plotted using Origin 8.1 (Electronic Arts Inc., Redwood City, CA, USA).

## 3. Results and Discussion

### 3.1. Characterization of SP and SPC

**Figure 1:**
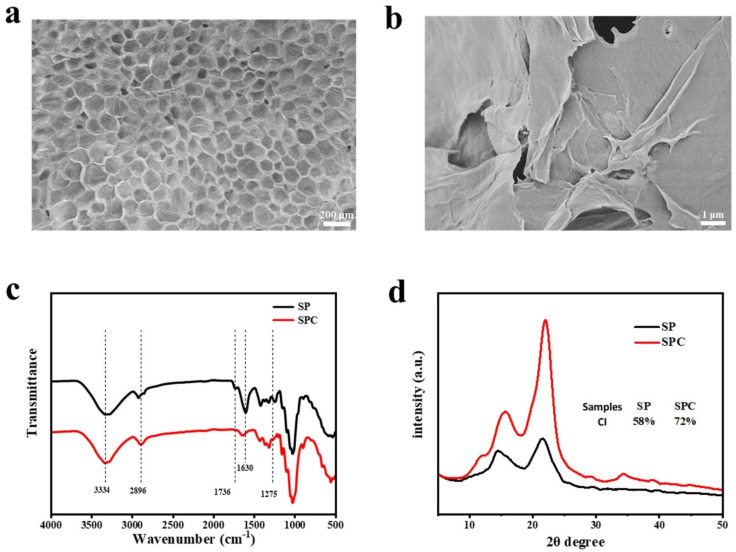
SEM images of (**a**) SP and (**b**) SPC, (**c**) FTIR spectra and (**d**) XRD patterns of SP and SPC.

The structure of sunflower straw primarily consists of an outer epidermis and an inner pith. SP is derived from fundamental meristematic tissue. It primarily comprises parenchyma cells with intercellular spaces (Figure S1). After drying, the SP exhibits a distinct honeycomb structure with a tetrahedral morphology (Figure 1a). Following purification and mechanical grinding, the pith cellulose transforms into a powder, and the SEM image of the SPC surface reveals a relatively smooth texture (Figure 1b).

As shown in Figure 1c, both SP and SPC exhibit characteristic vibrational absorption peaks at 1048 cm^−1^ and 3424 cm^−1^, corresponding to the C-O and -OH vibrational modes, confirming that cellulose is the primary constituent of both materials. Notably, SP shows a hemicellulose (C=O) stretching vibration peak at 1736 cm^−1^ and a strong absorption peak at 1630 cm^−1^, indicating the presence of lignin components. After purification, these two characteristic peaks disappear in the FTIR spectrum of SPC, confirming the effective removal of hemicellulose and lignin during purification.

Additionally, the XRD patterns of SP and SPC exhibit characteristic peaks of cellulose corresponding to the (110) and (200) planes at 14° and 22°, respectively (Figure 1d), indicating that the crystalline structure of cellulose remains consistent before and after purification. We calculated the crystallinity of SP and SPC using the Segal method [50], with results of 58% and 72%, respectively. The significant increase in crystallinity in SPC suggests that the purification process selectively removes amorphous components (i.e., hemicellulose and lignin) while preserving the inherent crystalline structure of cellulose [51].

The National Renewable Energy Laboratory (NREL) standardized protocols to characterize SP and SPC’s chemical composition and content. As shown in Table 1, the glucose content in SP was 31.52%, while the combined content of xylose and arabinose was 8.54%, and lignin content was 3.56%. These values indicate that SP’s hemicellulose and lignin content are relatively low, particularly lignin, which is significantly lower than other straw materials (Table 2). After purification, the glucose content in SPC increased to 89.54%, the xylose content was reduced to 4.70%, and the contents of other components were below 1%. During the alkaline soaking and bleaching process, hemicellulose and lignin were effectively removed, consistent with the observations in Figure 1. Thus, sunflower stalks, as a source of cellulose, offer the advantages of high cellulose content and low impurities, allowing for more efficient extraction and utilization of cellulose.

### 3.2. Humidity-Responsive SP-NP Film with Mechanical Response

When SP undergoes direct sulfuric acid hydrolysis, the amorphous regions in the cellulose are degraded, and partial dissociation of pectin, hemicellulose, and lignin also occurs. Sulfuric acid esterifies the hydroxyl groups, introducing charged sulfonate groups into the various components. After further ultrasonic treatment, the hydrolyzed SP transforms into a nanoscale aqueous SP-NP suspension containing nanocellulose, hemicellulose, and pectin.

**Figure 2:**
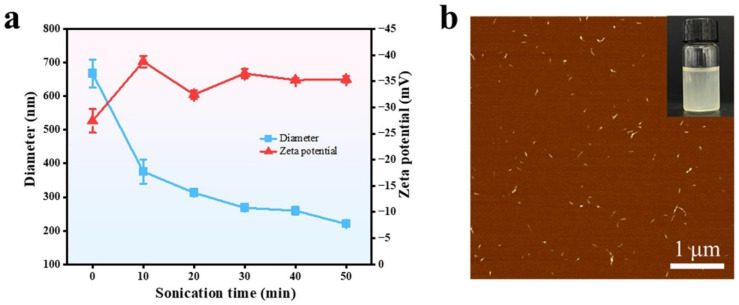
(**a**) zeta potential values and diameters of SP-NP prepared at different sonication times, (**b**) AFM image of SP-NP.

Figure 2a shows the zeta potential and average particle diameter of SP-NP prepared under different ultrasonic treatment durations. The cavitation effect induced by ultrasound disrupts the relatively weak hydrogen bonds between cellulose and other components, facilitating the breaking up of the nanoscale suspension. After 10 min of ultrasonic treatment, the particle diameter of the nanoscale suspension significantly decreased from an initial size of 667.5 ± 41.9 nm to 375.9 ± 35.8 nm. Simultaneously, the zeta potential measured also decreased from −27.4 ± 2.2 mV to −35.4 ± 0.5 mV due to the increased number of charged groups exposed on the surface of the suspension. With prolonged ultrasonic treatment, the zeta potential of the nanoscale suspension stabilized at approximately −35 mV, and the particle diameter continued to decrease gradually. Considering the balance between nano-suspension morphology and energy consumption, the sample treated for 30 min was selected for subsequent experimental procedures.

AFM was further employed to measure the morphological dimensions of SP-NP, and ImageJ software was used for quantitative analysis of its length and diameter. The results indicated that SP-NP sonicated for 30 min was stably dispersed as individual entities in the aqueous phase, with a morphology of short rods or particles (Figure 2b). Its length was 81.9 ± 1.3 nm, and its diameter was 4.7 ± 0.2 nm (Figure S2).

**Figure 3:**
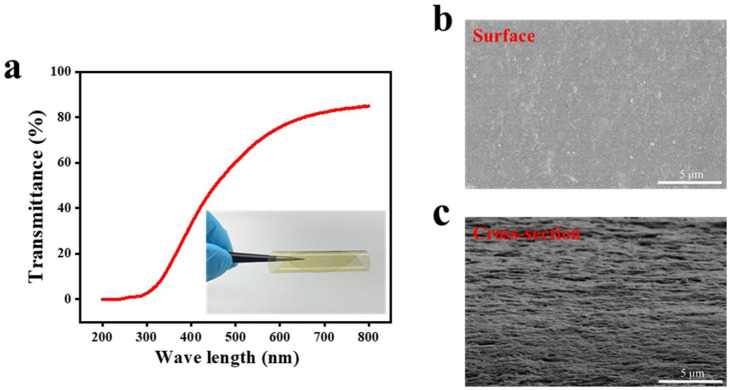
Characterization of SP-NP-30 film: (**a**) UV absorption and macroscopic view, (**b**) surface SEM, (**c**) cross-section SEM.

During the drying process at room temperature, nanoparticles or molecules dispersed in the aqueous phase gradually stack together under the influence of gravity as the water evaporates, forming hydrogen bonds that promote their tight binding and ultimately transform them into a stable film material. After ultrasonic treatment for 30 min, SP-NP spontaneously assembles into a film through drying. Due to the inhomogeneity and directional shrinkage during the drying process, an anisotropic stress distribution arises within the film, leading to continuous cylindrical curling along a specific direction. The film curls to reduce its internal stress levels, resulting in a fully rolled cylindrical shape. The obtained SP-NP film exhibits a light yellow appearance and has an average transmittance of over 60% within the visible light spectrum (Figure 3a). The film demonstrates excellent flexibility and remains intact after multiple bending and folding cycles.

To further investigate the microstructure of the film, liquid nitrogen was used to fracture the film through cryogenic breakage, and the surface and cross-section of the film were observed using SEM. Due to the high dispersibility and uniformity of the nanoscale suspension, the film surface remains highly smooth on the microscopic scale, with an axially aligned and uniform arrangement on the horizontal plane (Figure 3b). In the cross-sectional view, the film displays a distinct layer-by-layer stacked assembly structure with intermittent voids between the layers (Figure 3c). This dense but unevenly arranged structure results from the irregular hydrogen-bonding connections formed by the sporadic distribution of accessible regions between the nanofibers. The high-density regions between fibers correspond to the stress concentration areas of the film, while the voids represent defect zones.

Due to the primary compositions of the SP-NP film being cellulose, pectin, and hemicellulose, the abundance of hydroxyl groups imparts the film with inherently high hydrophilicity. The film rapidly transitions from a flat state to a U-shaped bend when placed directly on an exposed palm. However, when the film is placed on a gloved hand, it remains flat, exhibiting no deformation (Figure S3). This observation indicates that even the minimal moisture evaporating from the palm can induce significant deformation in the film, demonstrating its high sensitivity to humidity.

**Figure 4:**
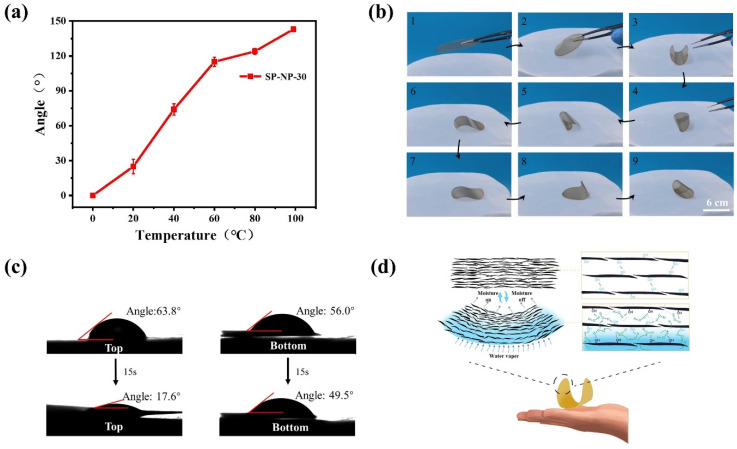
(**a**) Relationship between the bending angles of the SP-NP film and the temperature of the water in the beaker, (**b**) mechanical motion of the SP-NP film on moist nylon mesh, (**c**) water contact angles of the upper and lower surfaces of the SP-NP film at 0 s and 15 s, (**d**) mechanism of the mechanical motion of the SP-NP film under moisture gradient.

Deionized water was placed in a beaker covered with a breathable stainless-steel mesh, and the film was laid flat on the mesh to quantify the range of humidity-induced deformation. A glass plate was used to partially cover the film, after which the water in the beaker was gradually heated, and the degree of deformation of the film was observed. As the water temperature increased, the evaporation rate rose, creating a humidity gradient in the upper space of the beaker, which caused a significant increase in the bending angle of the film (Figure S4); the statistics of the bending angles are plot in Figure 4a. Additionally, when the film was placed on a partially wetted sponge, it rapidly exhibited instantaneous bending and demonstrated bidirectional bending during flipping (Figure S5). These results confirm the humidity-triggered actuation ability of the film and highlight its dynamic flexibility in deformation.

When the SP-NP film, assembled after 30 min of ultrasonic treatment (referred to as SP-NP-30 film), was placed in a beaker filled with 37 °C deionized water and covered with a nylon mesh, the film initially curled upward at both edges. It formed a cylindrical shape. Subsequently, the film began to roll due to a shift in its center of gravity, and the cylindrical curl gradually unfurled into an S-shaped deformation, causing the top and bottom surfaces to switch. The film then curled upwards at both edges, forming a cylinder, and rolled back towards its original position, unfurling once more and initiating a cyclic flipping motion (Figure 4b). The film displayed complex dynamic movements throughout this deformation process, including curling, rolling, and flipping. This sequence of coherent deformation movements underscores the high sensitivity and dynamic response of the film to humidity changes, revealing its potential for applications in humidity-driven actuation systems.

The hydrophilicity and moisture absorption properties of the upper and lower surfaces of the film were measured further to explore the humidity-responsive deformation mechanism. Given the marked hydrophilicity of the polysaccharide components, the water contact angles of the upper and lower surfaces were found to be 63.8° and 56.0°, respectively (Figure 4c). After 15 s, the degree of droplet absorption varied between the surfaces. The upper surface exhibited a faster penetration rate, with the contact angle decreasing to 17.6°, while the contact angle of the lower surface only decreased by 6.5°. This difference in the water absorption rate is likely related to the surface smoothness and the arrangement of the nano-dispersed particles. Analyzing the dynamic differences in water molecule adsorption and penetration makes it possible to design humidity-responsive sensors with specific response rates.

Based on the experimental data, we hypothesize that the bending behavior of the film in response to humidity changes may arise from asymmetric swelling. Specifically, when exposed to a humid environment, water molecules strongly interact with the abundant hydroxyl groups on the surface of the SP polysaccharide nanoparticles (Figure 4d). This process weakens the hydrogen bonding between the stacked nanofiber layers, promoting pore expansion and reducing tensile strength. Due to the tightly layered structure and the constraint of swelling, the film exhibits significant resistance to water vapor diffusion, slowing the uniform distribution of water molecules within the film. As a result, the film surface exposed to high humidity rapidly adsorbs water molecules and swells.

In contrast, the lower humidity surface remains relatively dry, creating a humidity gradient across the cross-section of the film. It leads to differential expansion across the thickness of the film, causing uneven internal stress distribution and subsequent bending deformation. Notably, once the water molecules are removed, the hydrogen-bonding network between the layers of the SP-NP-30 film reestablishes, allowing the film to return to its original unbent state. This reversible process highlights the unique advantages and potential applications of the SP-NP-30 film in humidity-driven deformation systems.

### 3.3. Humidity-Responsive SPC-NC Film with Color Response

**Figure 5:**
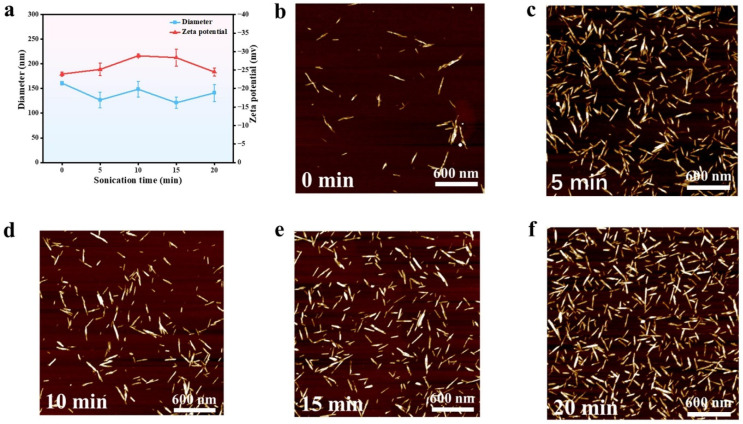
(**a**) Zeta potential values and diameters of SPC-NC prepared at different sonication times, AFM images of SPC-NC sonicated for (**b**) 0 min, (**c**) 5 min, (**d**) 10 min, (**e**) 15 min, and (**f**) 20 min.

Purified SPC was subjected to sulfuric acid hydrolysis and ultrasonic treatment to obtain a highly pure aqueous suspension of short rod-shaped SPC-NC. Previous studies have shown that cellulose nanocrystals (CNCs) can self-assemble into cholesteric liquid crystals (LCs) in aqueous solutions, a process regulated by factors such as the aspect ratio, concentration, and surface charge density of the CNCs [52,53]. Figure 5a illustrates the zeta potential and particle diameter of SPC-NC prepared with different ultrasonic treatment times. The ultrasonic treatment time increased from 5 to 20 min, during which the zeta potential of SPC-NC fluctuated between −23.9 mV and −28.8 mV. The highest absolute zeta potential was observed at 10 min, and the ultrasonic treatment time had a relatively small effect on the particle diameter of SPC-NC, which ranged between 100 and 150 nm. Overall, a 10 min ultrasonic treatment yielded a stable suspension of SPC-NC in aqueous solution.

Atomic force microscopy (AFM) characterization showed that SPC-NC exhibited a short rod-like structure dispersed in the aqueous phase (Figure 5b–f). Further quantitative analysis of the diameter and length of SPC-NC prepared with different ultrasonic treatment times revealed that, under ultrasonic treatment, the modified cellulose fractured along its axis, transforming into shorter rod-shaped SPC-NC, with the fiber length reduced to 118–126 nm (Figure S6). Meanwhile, no significant change in SPC-NC diameter was observed (Figure S7).

**Figure 6:**
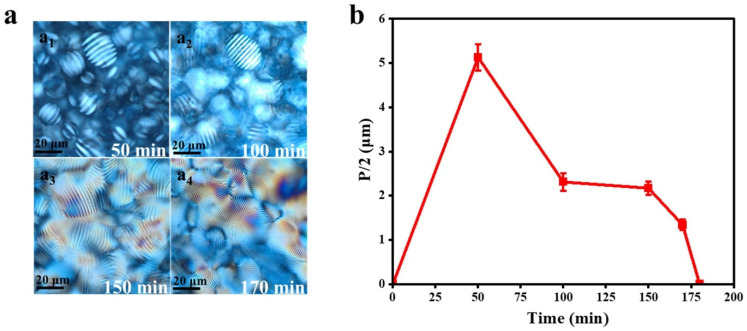
(**a**) Polarized optical microscopy images of SPC-NC suspensions at different assembly times, (**b**) relationship between the pitch of crystalloids and self-assembly time.

CNC are uniquely crystalline and exhibit remarkable cholesteric liquid crystal properties. A 1.0% SPC-NC suspension was subjected to 10 min of ultrasonic treatment and then added to a plastic petri dish. As the water naturally evaporated, the concentration of SPC-NC progressively increased. When the concentration exceeded the critical threshold, SPC-NC self-assembled into a chiral nematic structure with a spiral arrangement (Figure 6a). These chiral nematic domains grew, and the helical pitch gradually decreased (Figure 6b). As the distance between the individual chiral domains shortened, they merged, forming a long-range, ordered, unified chiral structure. Finally, during the late drying stages, these chiral nematic structures were fixed in place and transferred to the film material through hydrogen-bonding interactions.

**Figure 7:**
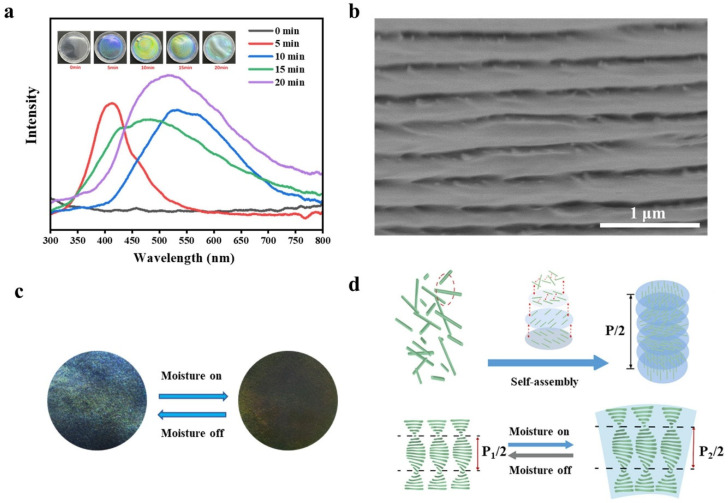
(**a**) Reflectance spectra and self-assembled structural colors of SPC-NC films at varying ultrasonication times, (**b**) SEM image of the cross-section of self-assembled SPC-NC film, (**c**) color transition of SPC-NC film at 99% RH, (**d**) mechanism of the humidity-induced color transition in SPC-NC films.

When the pitch of the helical cholesteric structure in the SPC-NC films falls within a specific range, the structure selectively reflects visible light, exhibiting structural coloration. Films were prepared by air-drying SPC-NC suspensions subjected to different durations of ultrasonic treatment. As shown in Figure 7a, due to the differences in the size and morphology of SPC-NC, the pitch of the self-assembled helical cholesteric structures varied, leading to diverse structural colors in the different films. With increasing ultrasonic treatment time, the wavelength of the reflected light progressively increased. As ultrasonic treatment time increased, the wavelength of the reflected light progressively shifted from 413 nm to 505 nm. Correspondingly, the structural color transitioned from bluish-purple to bright yellow. This shift is likely due to a decrease in the aspect ratio of SPC-NC caused by ultrasonic treatment, which leads to an increase in the pitch of the helical cholesteric structure. The cross-section of the SPC-NC film, after 10 min of ultrasonic treatment, was characterized by scanning electron microscopy (SEM) after fracturing in liquid nitrogen. Figure 7b reveals the uniformly ordered helical cholesteric structure of the cross-section.

The specific structural coloration of SPC-NC films is directly related to the pitch of the helical cholesteric structure. SPC-NC films are highly hydrophilic, absorbing water molecules from the air in high-humidity environments. This causes swelling and an increase in the pitch of the helical structure. When the SPC-NC film is wetted with water, its distinct blue structural color gradually disappears (Figure 7c), the gradually red-shifting spectral changes are shown in Figure S8. Figure 7d provides a schematic representation of the structural changes occurring in SPC-NC films during moisture absorption. When the film is exposed to a humid environment, SPC-NC absorb water, expanding the cholesteric structure and increasing the pitch (P). This increase in pitch causes a red shift in the wavelength of light selectively reflected by the film, eventually shifting beyond the visible range. Based on these properties, SPC-NC films show potential for applications in novel humidity sensors or visual indicators.

## 4. Conclusions

This study successfully developed a novel nanocellulose film material with significant humidity-responsive characteristics and structural color changes, utilizing sunflower pith (SP) as the raw material. Through sulfuric acid hydrolysis and ultrasonic treatment, we systematically analyzed the effects of different processing conditions on the morphology and size of sunflower nanofibers (SP-NP) and cellulose nanocrystals (SPC-NC). The results indicated that as the ultrasonic treatment time increased, the particle diameter of the nanocellulose gradually decreased, while its zeta potential remained between −30 mV and −40 mV, highlighting the critical role of ultrasonic treatment in precisely modulating the properties of nanocellulose. The nanocellulose film exhibited a unique layered stacking structure during the drying process, significantly enhancing its sensitivity and adaptability to changes in environmental humidity. The observed reversible deformation and mobility characteristics suggest promising applications in humidity-driven actuators and sensors. Moreover, the hydrolyzed cellulose nanocrystals spontaneously assembled into a chiral nematic structure, resulting in unique and striking structural color changes under varying humidity conditions, providing a new approach for the valorization of sunflower pith. This study also explored the complex molecular interactions between water molecules and SP-NP/SPC-NC, revealing the intrinsic mechanisms behind their humidity-responsive behavior. However, there are some limitations to the research, including challenges related to the collection and pretreatment of agricultural waste, consistency issues in the quality of sulfuric acid hydrolysis and ultrasonic treatment, and the long-term stability of the films under varying humidity conditions. Future research should focus on developing efficient and environmentally friendly methods for extracting and modifying nanocellulose to ensure the consistent quality of the films in large-scale production, as well as exploring their potential applications in emerging fields such as smart packaging and biomedicine. These efforts will further promote the practical application and industrialization of nanocellulose.

## Figures and Tables

**Table 1:** Chemical composition of SP and SPC.

Samples/ Ingredient	Glucose	Xylan	Arabinose	Acid-Soluble Lignin	Klason Lignin
SP (%)	31.52%	7.25%	1.29%	3.23%	0.33%
SPC (%)	89.54%	4.70%	0.01%	0.67%	0.70%

**Table 2:** Analysis of the composition of sunflower, sorghum stalks, and corn stalk pith [14].

Composition (%)	Sunflower Pith	Sorghum Stalk Core	Corn Stalk Core
Cellulose	47.4	35.0	24.6
Hemicellulose	9.40	34.5	19.1
Lignin	3.49	17.4	12.3
Hot water extractives	18.7	4.29	30.5
Total reducing Sugars	1.0	1.56	22.3
Ash content	20.4	4.23	5.19
Pectin	6.0	2.0	3.5
Benzene-alcohol Extract	4.91	4.51	5.09
Moisture	11.5	8.52	8.94

## Data Availability

The original contributions presented in the study are included in the article, and further inquiries can be directed to the corresponding author.

## Supplementary Materials

The following supporting information can be downloaded at: https://www.mdpi.com/article/10.3390/polym16223199/s1, Figure S1: Photographic depiction of structural characteristics; Figure S2: Size distribution of SP-NP after 30 minutes of ultrasonication; Figure S3: Proximal finger-induced upward bending response of nanocellulose film strips; Figure S4: Schematic diagram for quantifying the range of deformation induced by humidity; Figure S5: Bending phenomena of SP-NP-30 film on partially wetted sponge substrates; Figure S6: Length size distribution of SPC-NC under different sonication times; Figure S7: Diameter size distribution of SPC-NC under different sonication times; Figure S8: The reflection spectrum of the color change of the film.
