# Supplementary material for: Mechanical Motion and Color Change of Humidity-Responsive Cellulose Nanocrystal Films from Sunflower Pith
Source: Polymers (Basel). 2024 Nov 18;16(22):3199. doi: 10.3390/polym16223199 (PMC11598626; doi:10.3390/polym16223199)
Supplement: Supplementary file 1 [file polymers-16-03199-s001.zip › polymers-3250255-supplementary.pdf]

Mechanical motion and color change of humidity-responsive cellulose nanocrystal films from sunflower pith

Shujie Wang[1], Yanan Liu[1], Zhengkun Tao[1], Yang Li[1], Jie Jiang[2]*, Ke Zheng[1]*

1 Biomass Molecular Engineering Center and Department of Materials Science and Engineering, School of Materials and Chemistry, Anhui Agricultural University, 130 West Changjiang Road, Hefei, 230036, China; jiangdong412@stu.ahau.edu.cn (S.W.); liuyanan@stu.ahau.edu.cn (Y.Liu); 19856265392@163.com (Z.T.); 23720191@stu.ahau.edu.cn (Y.Li)

2 School of Resources and Environmental Engineering, Jiangsu University of Technology, Changzhou 213001, China

* Correspondence: zhengke@ahau.edu.cn (K.Z.); jiangjie@jsut.edu.cn (J.J.)

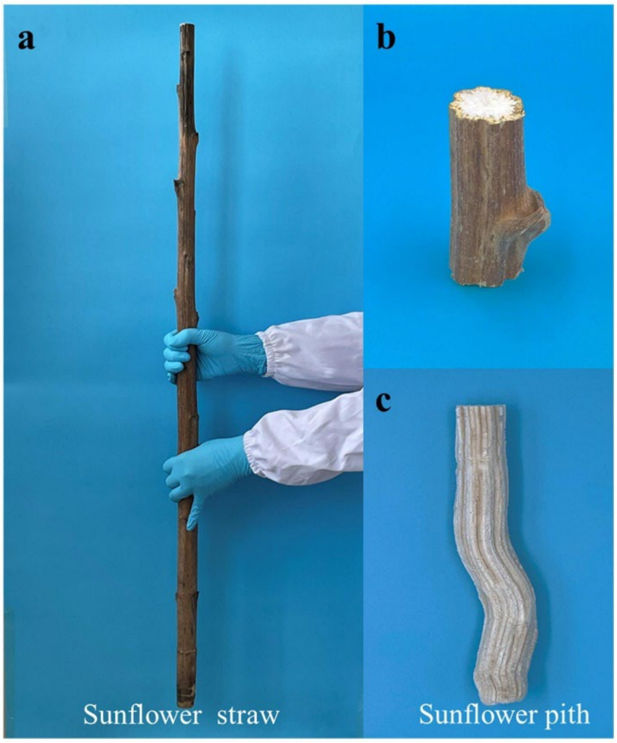

Figure S1 Photographic depiction of structural characteristics: (a, b) sunflower straw, (c) sunflower pith core.

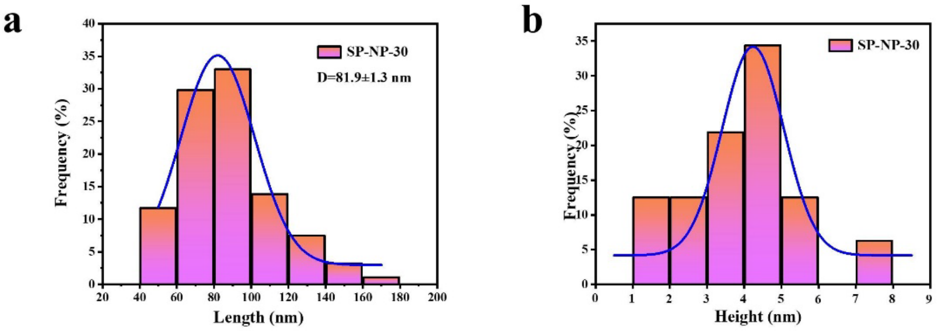

Figure S2 Size distribution of SP-NP after 30 minutes of ultrasonication: (a) length frequency, (b) diameter frequency.

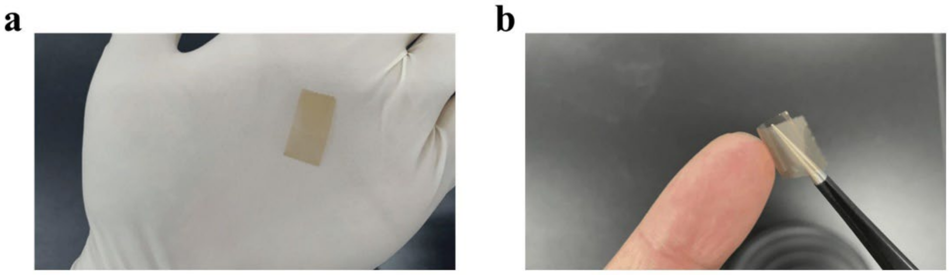

Figure S3 Proximal finger-induced upward bending response of nanocellulose film strips.

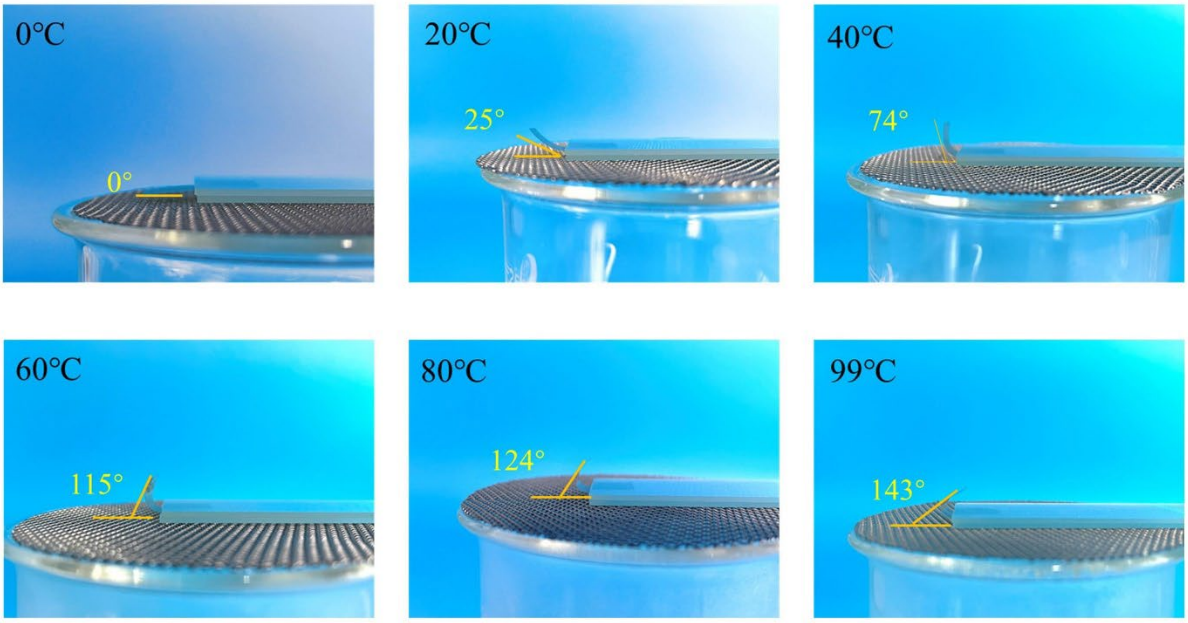

Figure S4 Schematic diagram for quantifying the range of deformation induced by humidity.

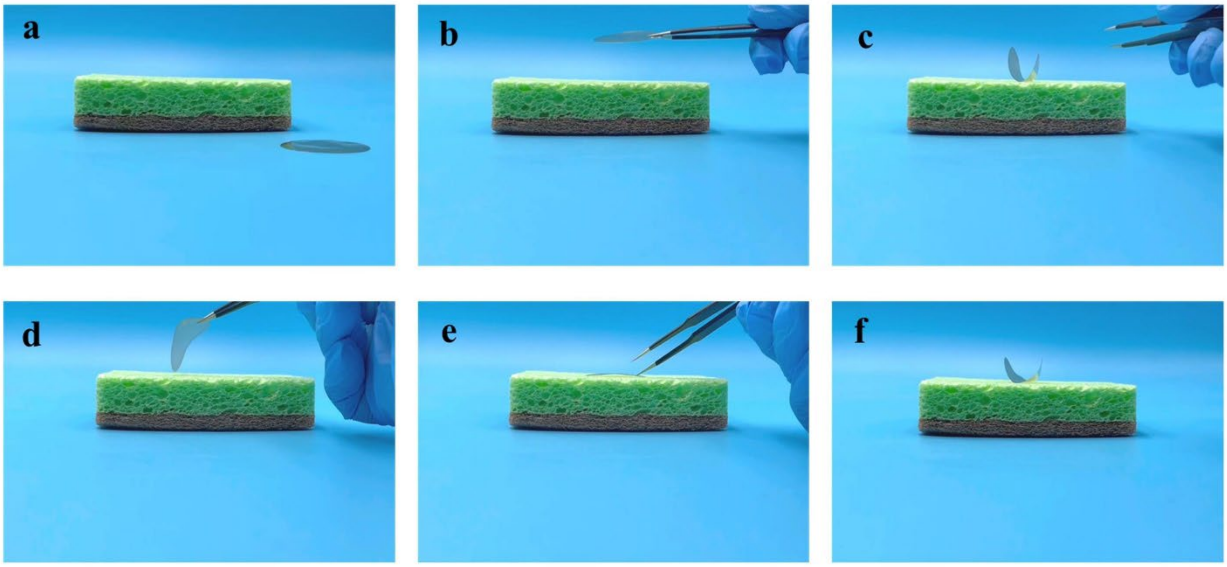

Figure S5 Bending phenomena of SP-NP-30 film on partially wetted sponge substrates.

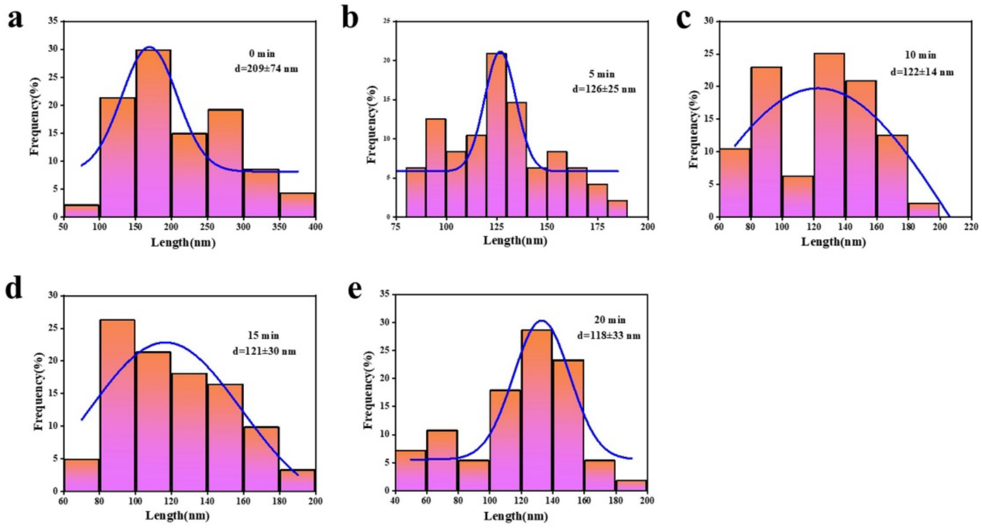

Figure S6 SPC-NCs size distribution by sonication time: length frequency at (a) 0, (b) 5, (c) 10, (d) 15, and (e) 20 minutes.

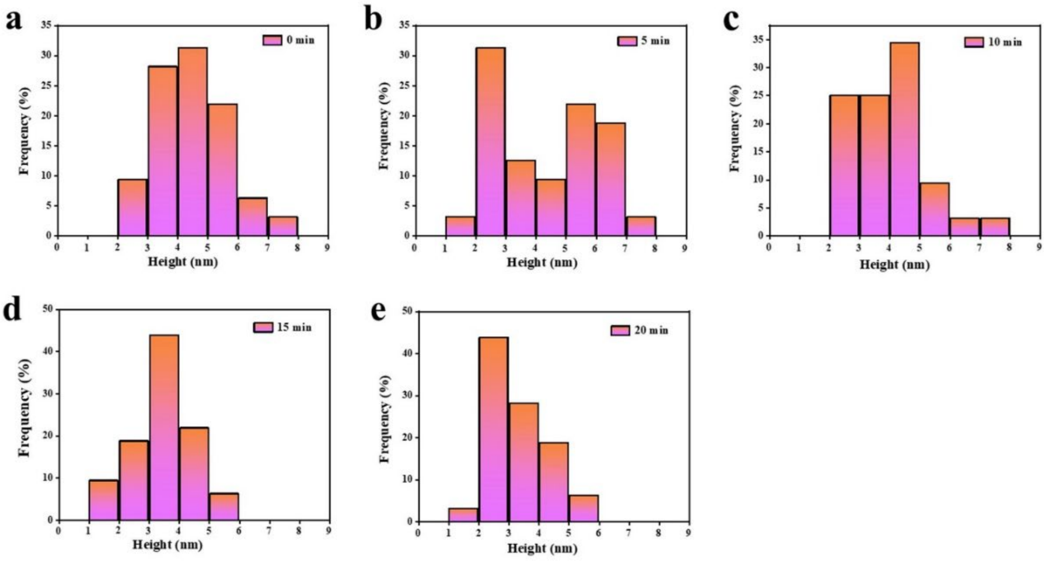

Figure S7 SPC-NCs size distribution by sonication time: height frequency at (a) 0, (b) 5, (c) 10, (d) 15, and (e) 20 minutes.

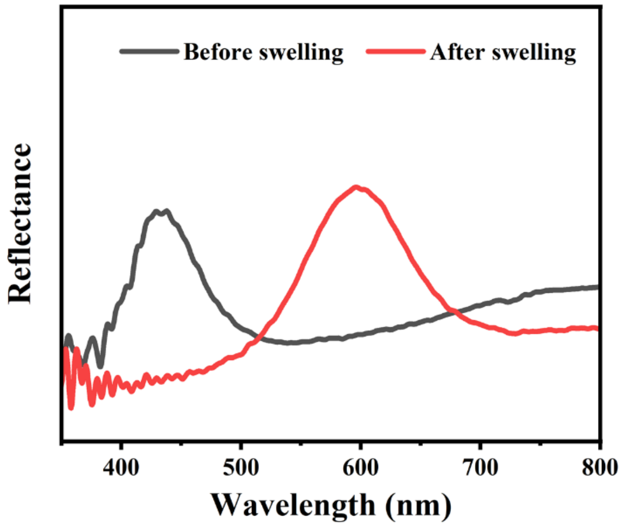

Figure S8 The reflection spectrum of the color change of the film.
